# Supplementary figures and images for: Effects of TMS on the Decoding and Electrophysiology of Priority in Working Memory
Source: eNeuro. 2026 Apr 21;13(4):ENEURO.0346-25.2026. doi: 10.1523/ENEURO.0346-25.2026 (PMC13120838; doi:10.1523/ENEURO.0346-25.2026)

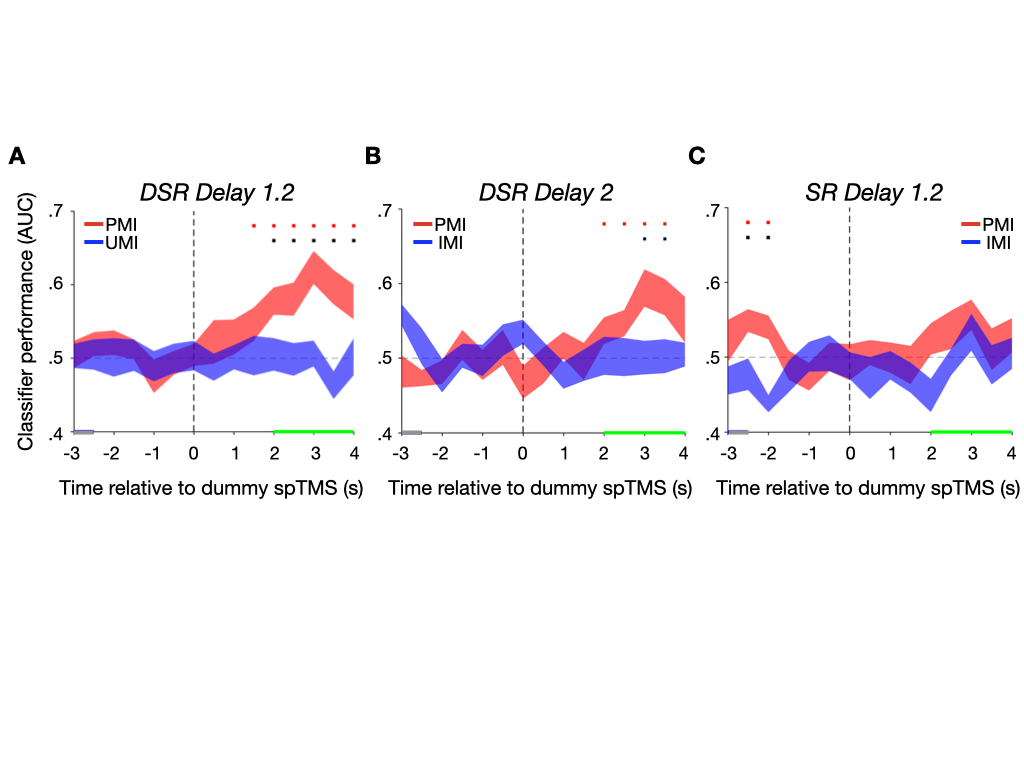

Supplement: Figure 3-1 — Broadband power classifier performance for trials without spTMS. A. Classifier AUC during the DSR Delay 1.2 epoch for the prioritized memory category (PMI) in red and the unprioritized/irrelevant memory category (UMI/IMI) in blue. Gray highlighting along the x-axis corresponds to the retrocue period, green highlighting corresponds to the probe + feedback period, with the delay period occurring between and the spTMS pulse time-locked to 0 seconds. B. Same as A. for DSR Delay 2. C. Same as A. & B. for SR Delay 1.2. Error bands correspond to +/– 1 standard error of the mean for the n = 12 participants. Red significance squares correspond to significant AUC clusters for PMI decoding; blue squares correspond to significant AUC clusters for UMI/IMI decoding; black squares correspond to significant AUC PMI-UMI/IMI difference clusters. Filled markers: p < .05; empty markers: p < .1. Blue * markers: p < .05 based on cluster-based permutation test results in A.-C. Download Figure 3-1, TIF file. [file eneuro-13-ENEURO.0346-25.2026-s002.tif]

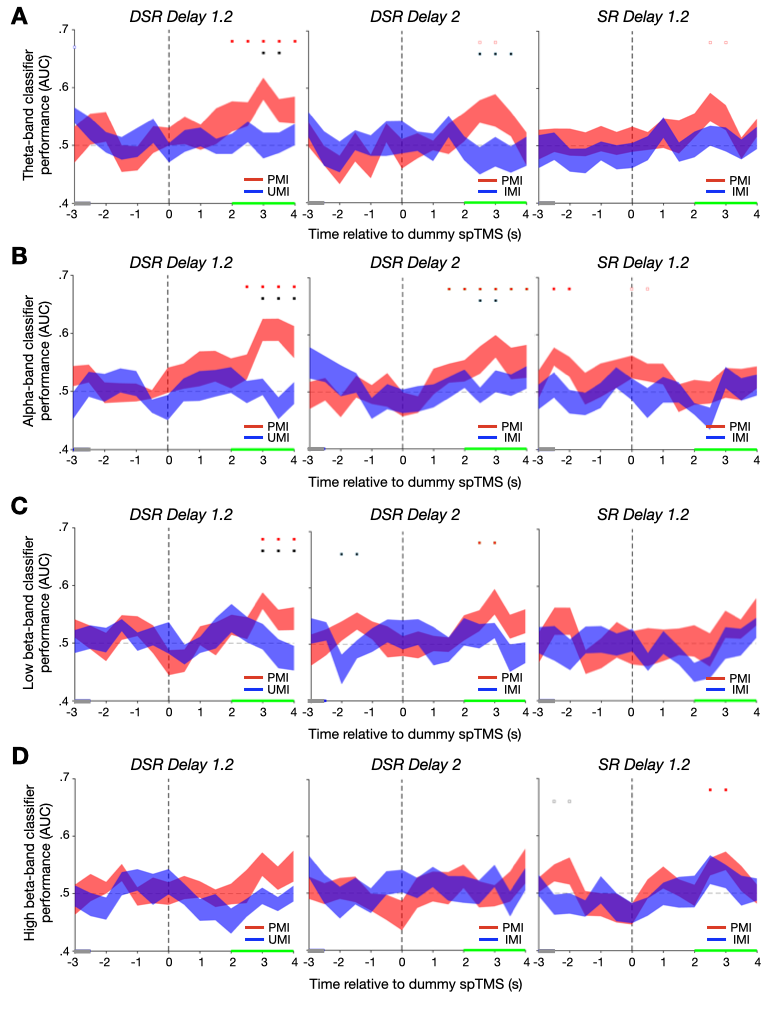

Supplement: Figure 4-1 — Frequency-band specific classifier performance for trials without spTMS delivery. A. Theta-band Classifier AUC during the DSR Delay 1.2 epoch (left), DSR Delay 2 (middle), and SR Delay 1.2 (right) for the prioritized memory category (PMI) in red and the unprioritized/irrelevant memory category (UMI/IMI) in blue. Gray highlighting along the x-axis corresponds to the retrocue period, green highlighting corresponds to the probe + feedback period, with the delay period occurring between and the spTMS pulse time-locked to 0 seconds. B. Same as A. for alpha-band classifier AUC. C. Same as A. & B. for low beta-band classifier AUC. D. Same as A., B., & C. for high beta-band classifier AUC. Error bands correspond to +/– 1 standard error of the mean for the n = 12 participants. Red significance squares correspond to significant AUC clusters for PMI decoding; blue squares correspond to significant AUC clusters for UMI/IMI decoding; black squares correspond to significant AUC PMI-UMI/IMI difference clusters. Filled markers: p < .05; empty markers: p < .1. Download Figure 4-1, TIF file. [file eneuro-13-ENEURO.0346-25.2026-s003.tif]

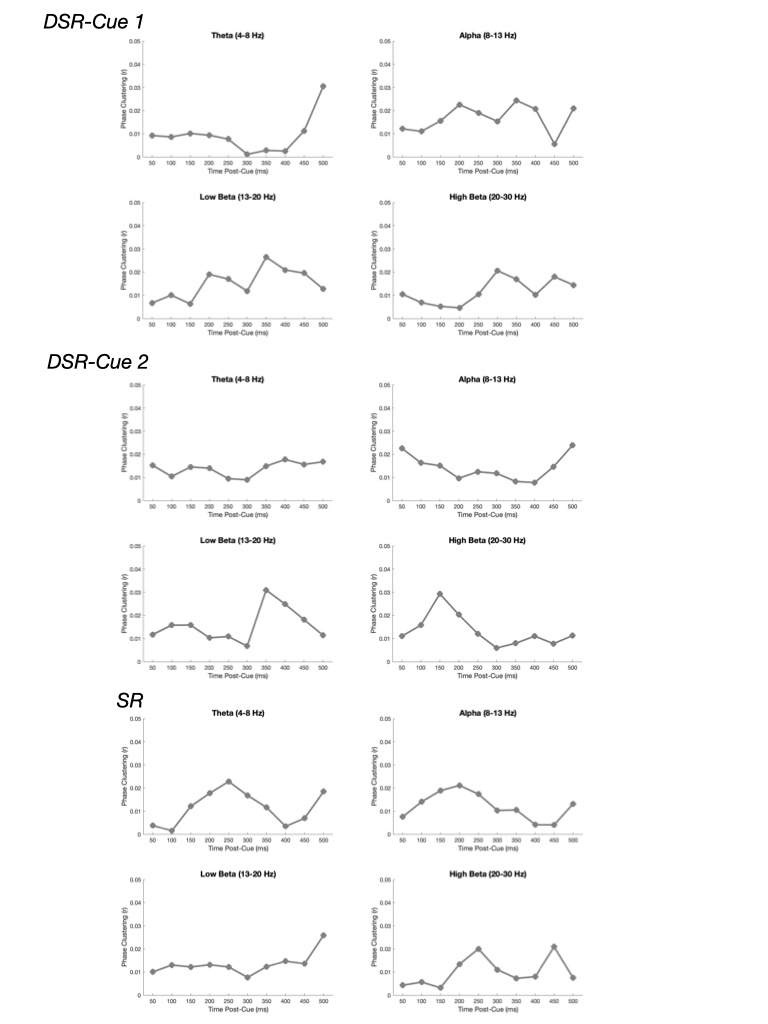

Supplement: Figure 6-1 — Time-resolved post-cue phase consistency analysis. Post-cue phase consistency between subjects at 10 timepoints 50 ms apart for each of the four frequency bands and three retrocues. None of the consistencies reached significance after FDR correction. Download Figure 6-1, TIF file. [file eneuro-13-ENEURO.0346-25.2026-s004.tif]

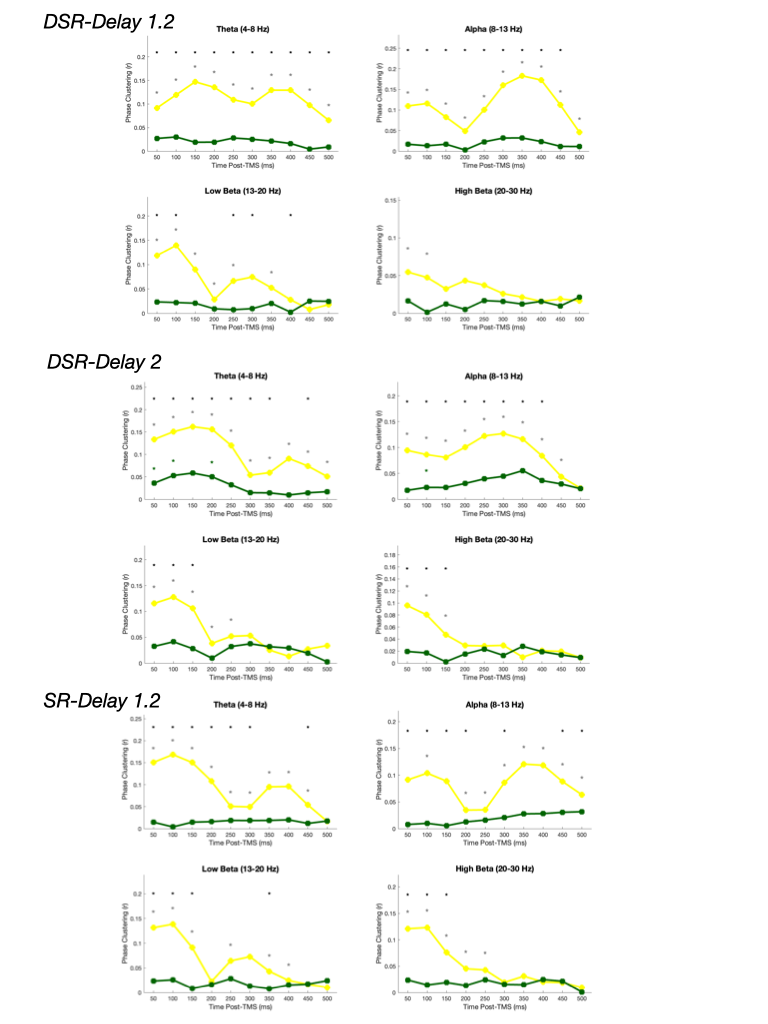

Supplement: Figure 6-2 — Time-resolved post-spTMS phase consistency analysis. Post-spTMS phase consistency between subjects (yellow) at 10 timepoints 50 ms apart for each of the four frequency bands and three retrocues. For comparison, post-dummy spTMS phase consistency is plotted in green. Significant FDR-corrected within-condition phase consistencies are indicated by gray * markers; significant FDR-corrected between condition phase consistencies are denoted by black * markers across the top of the plot. Download Figure 6-2, TIF file. [file eneuro-13-ENEURO.0346-25.2026-s005.tif]

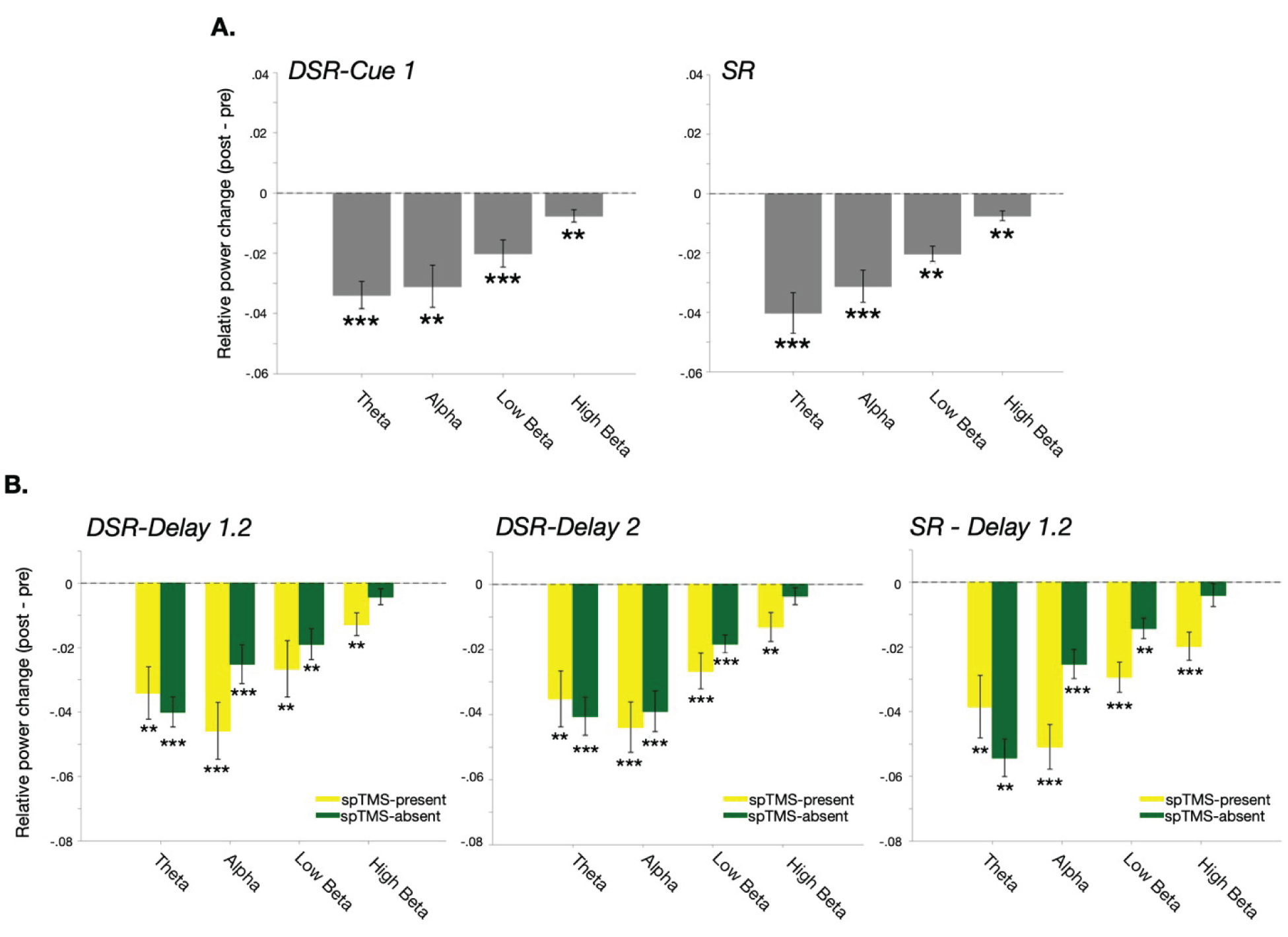

Supplement: Figure 6-3 — Within-subject power. A. Cue-evoked changes in relative power (post – pre) for each of the four frequency bands in response to Cue 1 (DSR; left) and the single cue in the SR task (right). B. Same as A. for spTMS-evoked changes of relative power, with comparison to baseline in trials when spTMS was not delivered. *** markers: p < .001; ** markers: p < .01; * markers: p < .05 based on permutation tests; error bars correspond to +/- 1 SEM. Download Figure 6-3, TIF file. [file eneuro-13-ENEURO.0346-25.2026-s006.tif]
